# Transgelin-2 Involves in the Apoptosis of Colorectal Cancer Cells Induced by Tanshinone-IIA

**DOI:** 10.1155/2022/9358583

**Published:** 2022-09-27

**Authors:** Yingru Zhang, Yiyang Zhao, Jingwen Liu, Chunpu Li, Ying Feng, Shasha Jiang, Xiaoting Sun, Xueqing Hu, Yan Wang

**Affiliations:** ^1^Department of Medical Oncology, Shuguang Hospital, Shanghai University of Traditional Chinese Medicine, Shanghai 201203, China; ^2^Academy of Integrative Medicine, Shanghai University of Traditional Chinese Medicine, Shanghai 201203, China

## Abstract

Tanshinone IIA (TanIIA) is the main active ingredient in the fat-soluble components isolated from *Salvia miltiorrhiza* Bunge. Our previous studies have convincingly proved that TanIIA is an effective drug against human colorectal carcinoma cells. In order to further demonstrate the effect of TanIIA on CRC, we carried out exploratory research about it in vivo and in vitro. The results demonstrated that TanIIA were observably more effective than control group in preventing tumor growth, and it has increased the survival time. Cancer cells viability and proliferation were accompanied by concentration and time dependent decline reached with TanIIA. We found that TanIIA altered the morphology of cytoskeleton and it could obviously induce apoptosis of colorectal cancer cells and block the cells in the G0/G1 phase. TanIIA also increased phosphorylation of p38MAPK, upregulated ATF-2 expression and downregulated Transgelin-2 expression, which could be reversed by SB203580, a p38MAPK-specific inhibitor. Our results suggested that TanIIA could induce apoptosis of colorectal cancer and block the cells in G0/G1 phase involved in downregulating the expression of Transgelin-2 through p38MAPK signal pathway.

## 1. Introduction

Colorectal cancer (CRC) is the third most common malignant tumor, during 2021 in United States, estimated 149,500 new cases were diagnosed and 52,980 deaths for CRC [1]. At present, the treatment of primary CRC is surgical resection [2]. For patients who cannot tolerate a surgery or bear a high risk of recurrence and metastasis, treatments include systemic and local chemotherapy, targeted agents and immunotherapy [3, 4]. What is more, many effective components extracted from traditional herbs exert evident antitumor effects and are expected to develop some specializing targeted drug in cancer development, creating a new field of cancer treatment [5, 6].

Tanshinone IIA (TanIIA) is a phenanthrene-quinone derivative isolated from Dan-shen which with a scientific name of *Salvia miltiorrhiza* Bunge, it has been used in the treatment of multiple diseases such as coronary heart diseases [7], metabolic disorders [8], pulmonary fibrosis [9], cancers [10], and inflammatory diseases [11], and it is expected to be relatively safe with regard to its toxicity. *Salvia miltiorrhiza* Bunge, a traditional Chinese medicine that has the effects of promoting blood circulation and removing blood stasis, menstruation and relieving pain [12]. *Salvia miltiorrhiza* Bunge mainly has two kinds of active components, one is hydrophilic components, such as Salvianolic acid, the other is lipophilic components, mainly tanshinones, such as tanshinone IIA and cryptotansetron (CPT) [13]. Current studies have confirmed that tanshinone, the key component of *Salvia miltiorrhiza* Bunge, has anticancer effect [14]. Tanshinone IIA, as the main anticancer component of *Salvia miltiorrhiza* Bunge, is rich in *Salvia miltiorrhiza* root and has a higher content than other components [15].

Our previous studies have firmly demonstrated that TanIIA could regulate epithelial-to-mesenchymal transition (EMT) to suppress the metastasis in CRC [16]. However, the molecular mechanism of its antitumor activity is not fully understood and deserves further investigation.

Transgelin, a family of actin-binding proteins, altering the structure and morphology of the cytoskeleton [17], and is associated with cell biology of cancer, including cell cycle, morphogenesis, and migration [2]. These proteins have the role of regulating proliferation and apoptosis in many different carcinoma cells including colorectal carcinoma cells [18, 19].

In this study, we further demonstrated that the apoptosis induction and proliferation inhibition of human colorectal cancer cells SW620 were highly correlated with the expression of Transgelin-2 via activating the p38MAPK pathway to regulate cell cycle. It will be helpful to provide experimental basis for the mechanisms of TanIIA in the treatment of CRC.

## 2. Materials and Methods

### 2.1. Experimental Animals

Healthy BALB/c nude mice were provided by the Experimental Animal Department of Shanghai University of Traditional Chinese Medicine for pharmacodynamic research. All the mice were provided a standard diet and placed in temperature-controlled facilities till the end of the study. All experiments were conducted according to the recommendations and approval of local animal protection legislation and the Ethics Committee is responsible for animal research.

### 2.2. Cell Lines

Human colorectal cancer cell line SW620 was bought in the Shanghai Biological Science and Technology limited company. Cells were cultured in L-15 (Corning, China) medium supplemented with 10% fetal bovine serum (Gibco Life Technologies, Grand Island, NY) and 1% penicillin and streptomycin (Gibco Life Technologies, Grand Island, NY) in 5% carbon dioxide-air of 37°C humidified incubator.

### 2.3. Drugs

TanIIA, purity >98%, purchased from Aladdin Biotechnology Co., Ltd; serial number: T109794; SB203580, purchased from MCE; serial number HY-10256A.

### 2.4. Animal Model

One hundred male nude mice (18-20 g, 5 weeks old) were subcutaneously injected with 2 × 10^6^ SW620 cells. One week later, mice were divided into five groups randomly and were, respectively, administered with normal saline (NS, 25 mL/kg), dimethyl sulfoxide (DMSO, 0.2%), or TanIIA solution at low (0.5 mg/kg)/middle (1 mg/kg)/high (2 mg/kg) dose, by injection through tail vein for 14 days at 1 vic/day. Ten mice of each group were observed the growth rate of tumors. When mice were sacrificed, all tumors were resected and examined by measuring the longest (a) and the shortest (b) vertical dimensions. Other ten mice in each group were used to observe their survival time, according to the ethical requirements, mice were humanely killed when the tumor volume reached 2000 mm^3^. The tumor size (V), growth rate (GR), and Survival rate (SR) were calculated according to following formulae: *V* = ab2/2, GR = (GTtest − GTcontrol)/GTcontrol × 100%, and SR = (STtest − STcontrol)/STcontrol × 100%.

### 2.5. TUNEL Assay

The TUNEL assay was detected with the In Situ Cell Death Detection Kit (Roche, Switzerland). The tumor tissues paraffin-embedded sections were deparaffinized in xylene, then added in a graded series of ethanol (Absolute, 95%, 75%, 5 min) and ruptured the cell membranes with 0.1%TritonX-100 (Sinopharm, China) for 8 min. Followed, the slides were rinsed in TBS three times and incubated with Equilibration Buffer for half an hour at room temperature. After that, the sections were incubated with Recombinant TdT Enzyme (Thermo Fisher, USA) in dark for 1 hour at 37°C. After incubation, the slides were washed with TBS 3 times (5 min), then incubated with DAPI at room temperature for 10 minutes, installed with antifade solution (Solarbio, S2100, Beijing), and stored at 4°C in the dark. Images were photographed at fluorescence microscope (Leica, Germany).

### 2.6. CCK-8 Assay

Human colorectal cancer cells SW620 were maintained in L-15 medium supplemented with 10% heat-inactivated fetal bovine serum. Experiments were performed at the log phase cell growth period. Cells were prepared at a concentration of 1 × 10^5^ /ml, and vaccinated at 100 *u*l/well in the 96-well plates, then either treated with DMSO, or with TanIIA at a series of concentration (0,2,4,8,16,32,64 *μ*mol/L) for 24, 48, and 72 hours, each with six replicates. A blank control group was set with no cells. Cell viabilities were assessed by CCK-8 assay after treatment with medium containing 10% CCK-8 reagent for 2 h. Measuring absorbance at 450 nm with microplate reader, and the cell viability was determined by dividing average reading of the treated well by the average reading of the control well. Straight-line correlation analysis was used to fix dosage and cell viability.

### 2.7. Colony Formation Assay

SW620 cells were seeded in 6-well plates at a density of 1000 cells per well. When cells attached, they were treated with different concentrations of TanIIA (1 *u*mol, 2 *u*mol, and 4 *u*mol). Medium was changed every 4 days. After 10 days incubation, cells were fixed and stained. Colonies composed of >50 cells were counted and photographed.

### 2.8. Hoechst 33258 Staining

The cells were cultured on the sterile cover glasses placed in 6-well plates overnight. After 2 days treatment by TanIIA, fixed and washed the cells twice and then stained with 1 mg/ml Hoechst 33258 for 5 minutes in darkness at normal temperature. Followed, washed the cells twice with PBS and photographed by fluorescence microscope (Leica, Germany, DM2500) with a 40 × objective lens. Apoptotic cells were identified by nuclear condensation and/or fragmentation.

### 2.9. Immunofluorescence Experiment

Briefly, when cell density reached 50% in 24-well plates, fixed with 4% paraformaldehyde, ruptured the cell membranes with 0.1% TritonX and the 45 min is sealed with 5%BSA, incubated with Phalloidin (Thermo Fisher, USA) in the dark at 37°C for 1 h, then washed by PBS 3 times (3 min), followed by incubation with DAPI for 5 min in the dark. At last, cells were mounted the slides with antifade solution. Images were taken by laser scanning confocal microscope (Leica, Germany).

### 2.10. Flow Cytometry Analysis

SW620 cells were cultured to exponential phase of growth and treated with serum-free culture medium for 12 h to synchronize the cell cycle. Then treated with different concentrations of TanIIA (4 *u*mol/L, 8 *u*mol/L, and 16 *u*mol/L) for 48 h, resuspended into single cell suspension and stained with anti-Annexin V FITC and PI. The influence of TanIIA on apoptosis and necrotic rate was examined by flow cytometry. Typically, early apoptotic cells present Annexin+/PI-, late apoptotic and necrotic cells present Annexin+/PI+, and normal cells present Annexin-/PI-, respectively.

### 2.11. Western Blot Analysis

The protein samples were transferred to the PVDF membrane after 10% SDS-polyacrylamide gel electrophoresis. The membrane was blocked in TBS-T blocking buffer containing 5% skim milk powder (10 mmol/L Tris, pH 7.5, 100 mmol/L NaCl, and 0.1% Tween 20) for 2 hours, prior to be incubated overnight at 4°C with specific antibodies against total p-p38MAPK, p38MAPK, ATF-2, Transgelin-2, Bcl2, Bax, GAPDH, and *β*-Actin antibody (CST, USA). The membrane was then incubated with a secondary goat antibody (beyotime, China) coupled with horseradish peroxidase (HRP) at room temperature for 2 hours, washing with TBS-Tween solution three times, and add AB developer (1 : 1, v/v).

### 2.12. Statistics and Analysis

Statistical analysis using SPSS 22.0 statistical software package for the (IBM, Chicago, IL, USA). One-way ANOVA was used for statistical analysis, and then Fisher minimum significant difference test was performed. All the results are expressed by the mean ± SE, *n* = 3, and a value of *P* < 0.05 was considered significant.

## 3. Results

### 3.1. TanIIA Inhibits the Growth of Colorectal Cancer and Induces the Apoptosis in Subcutaneous Xenograft Mouse Model

**Figure 1:**
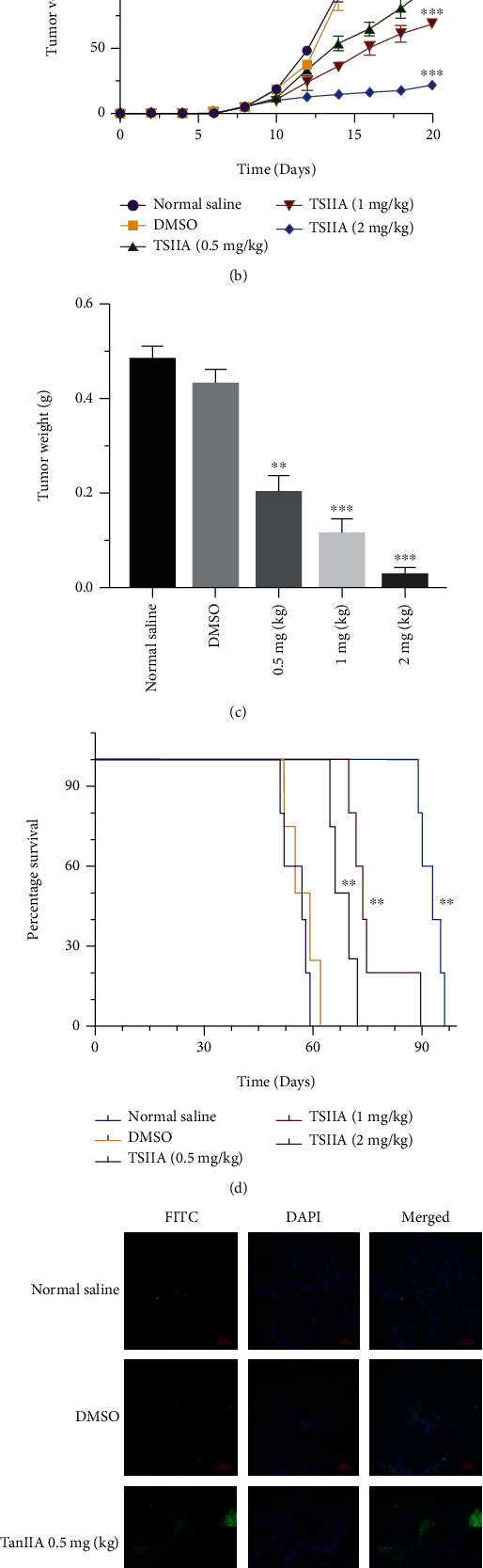
TanIIA inhibited the growth and proliferation of human colorectal cancer cell in vitro. (a) Tumor photos were taken from tumor-bearing nude mice after intravenous injection of normal saline, DMSO and TanIIA with 0.5 mg/kg, 1 mg/kg, 2 mg/kg for 2 weeks, respectively, and measured the volume (b) and the weight (c) of tumor. (d) Survival time of tumor-bearing nude mice compared with normal saline group after different treatment, ^∗∗^*P* < 0.01, ^∗∗∗^*P* < 0.001. (e) TUNEL assay was detected the degree of cell apoptosis in tumor-bearing nude mice, terminal deoxynucleotidyl transferase (green) bound to expose 3′-OH ends of DNA fragments generated. Apoptotic cells (TUNEL-positive cells) exhibit green fluorescence, and DAPI (blue fluorescence) stands for nucleus.

As shown in Figures 1(a)–1(c), the tumor volume inhibitory rates were positively correlated with the concentration of TanIIA, and the weights of colorectal tumor in nude mice were significantly reduced as approximately 90% after TanIIA high dose treatment compared to normal saline control group and DMSO group. In addition, the effect of TanIIA on the survival time of mice with colorectal tumor was also determined (Figure 1(d)). Fifty mice eventually died of colorectal cancer. Among them, TanIIA at high dose (2 mg/kg) showed the best prospect of improving the survival time of mice, up to 96 days (*P* < 0.01), while the survival time of the control group was 54 days. In the aspect of life prolongation rate, the effective rate of TanIIA group (2 mg/kg) was nearly twice as high as that of control group. Furthermore, TUNEL assay (Figure 1(e)) showed TanIIA treated group performed stronger staining than control, which indicated that TanIIA inhibits the growth of colorectal cancer and induce the apoptosis in vivo.

### 3.2. TanIIA Inhibits the Growth and Proliferation of Human CRC Cells In Vitro

**Figure 2:**
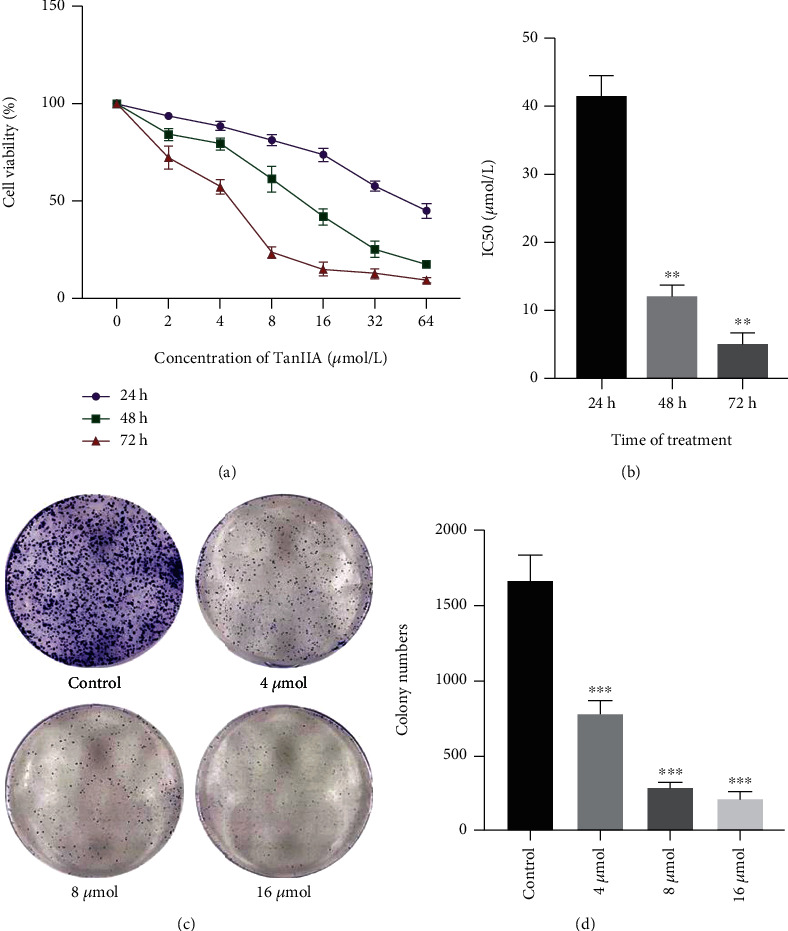
Effects of TanIIA on SW620 cell growth and proliferation. (a) Cell viability was evaluated by CCK assay after exposure to the 0, 2, 4, 8, 16, 32, and 64 *μ*mol/L concentrations of TanIIA for 24, 48, and 72 h. (b) The median inhibitory concentration (IC50) at 24, 48, and 72 h was 41.60, 11.76, and 5.289 *μ*mol/L, respectively, ^∗∗^*P* < 0.01, compared with 24 h. (c, d) The ability of cell proliferation was detected after TanIIA treatment by colony formation assay. ^∗∗∗^ *P* < 0.001, compared with control group.

For all the exposure time tested (24 h, 48 h, and 72 h), TanIIA treatment demonstrated significant inhibition effect of SW620 cells growth compared to control group (*P* < 0.01) in a dose-dependent manner (Figure 2(a)). The 50% inhibitory concentration (IC50) of TanIIA for 24, 48, and 72 h were 41.60, 11.76, and 5.289 *u*mol/L, respectively (Figure 2(b)). Clearly, higher concentrations of TanIIA produced significant growth inhibition within 24 h, and the effect was also in a time-dependent manner. Moreover, the colony formation test showed that the number of colony formation decreased with the increasing concentration of TanIIA (Figures 2(c) and 2(d)). All results indicated that TanIIA inhibits the growth and proliferation of human CRC cells in vitro.

### 3.3. TanIIA Induced Apoptosis and Cytoskeleton Changes in Colorectal Cancer Cells

**Figure 3:**
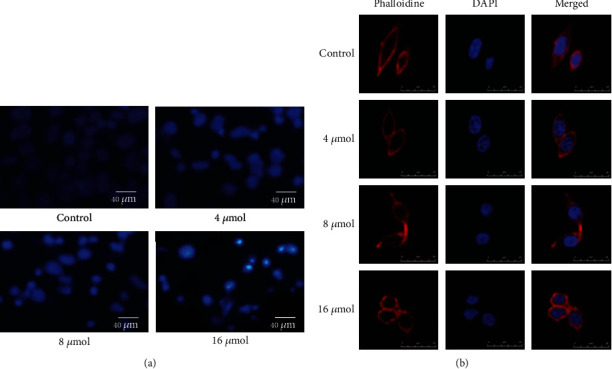
Morphological changes of SW620 cells induced by TanIIA. After cells treated by different concentrations of TanIIA (4 *u*mol, 8 *u*mol, and 16 *u*mol), cell nucleus stained with Hoechst 33258 (a) and cytoskeleton labeled with phalloidine (b).

Nuclear morphology was evaluated by membrane permeability blue Hoechst 33258. Under the fluorescent microscope, SW620 cells after treatment with TanIIA for 48 h showed apoptosis characterized morphological changes (Figure 3(a)). In control cultures, nuclei of SW620 cells appeared normal, round, and large in size. In contrast, most nuclei of SW620 cells treated with TanIIA had bright staining, small volume, and condensed chromatin. We found that the number of apoptotic nuclei containing condensed chromatin increased markedly with concentration dependent. Furtherly, to evaluate the morphological characteristics of cells, phalloidin labeled cells were observed with laser confocal microscopy.  Figure 3(b) showed that treatment with 16 *u*mol/L TanIIA for 24 h, cell-cell junction fell off and cytoskeleton shrank, with the cell bodies rounded, the volume reduction, karyopyknosis, and nuclear fragmentation. It was indicated that TanIIA induced colorectal cancer cell apoptosis and altered cytoskeleton.

### 3.4. Apoptosis Induced by TanIIA via Activating the p38 MAPK Pathway

**Figure 4:**
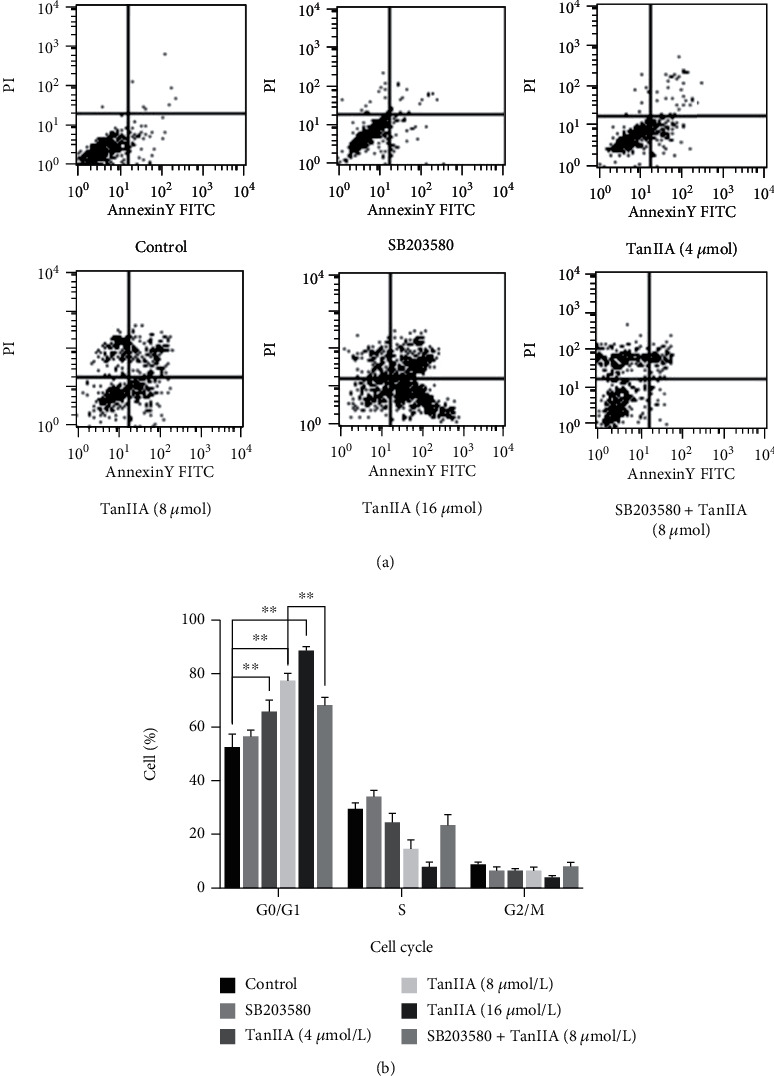
Apoptosis induced by TanIIA in SW620 cells. (a) Effects of TanIIA on early apoptotic events and late apoptotic/necrotic events of SW620 cells after treated with SB203580, TanIIA (4 *u*mol/L), TanIIA (8 *u*mol/L), TanIIA (16 *u*mol/L), and TanIIA (8 *u*mol/L) + SB203580 for 48 h. (b) The cell cycle percentage was evaluated by FACS, ^∗∗^*P* < 0.01.

The type of cell death is classified as apoptotic cell death or necrotic cell death, which can be detected by Annexin V-FITC and propidium iodide (PI) staining and flow cytometric analysis. Normal cells are Annexin V-/PI-, while cells in early apoptosis are Annexin V+/PI-, and cells in late apoptosis/necrosis are double positive. In our experiment (Figure 4(a)), the decrease of cell viability induced by TanIIA after 48 h treatment was combined with an increase in the number of cells that experienced early or late apoptotic events, and the increasement was significant compared to control, especially the proportion of cells remained in G0/G1 period increased, as shown in Figure 4(b). The cell apoptotic rates were (10.23 ± 2.05)%, (19.96 ± 3.47)%, and (37.08 ± 6.87)%, respectively, when treated with 4, 8, 16 *u*mol/L of TanIIA for 48 h.

Besides, after p38MAPK signaling pathway was blocked (treated with p38MAPK specific inhibitor SB203580 and TanIIA), the apoptosis rate and of G0/G1 phase cell ratio decreased significantly compared to TanIIA treated group. While the apoptosis rate and of G0/G1 phase cell ratio had no change compared to the group only treated with SB203580 (Figure 4(a)).

The results indicated that TanIIA could induce significant colorectal cancer cell apoptosis in SW620 cells after treatment with TanIIA for 48 h and block the cells in G0/G1 phase in a dose-dependent manner. p38MAPK signaling pathway may play a role in TanIIA-induced apoptosis of SW620 cells.

### 3.5. The Expression of Transgelin-2 Decreased with the Activation of p38 MAPK by TanIIA

**Figure 5:**
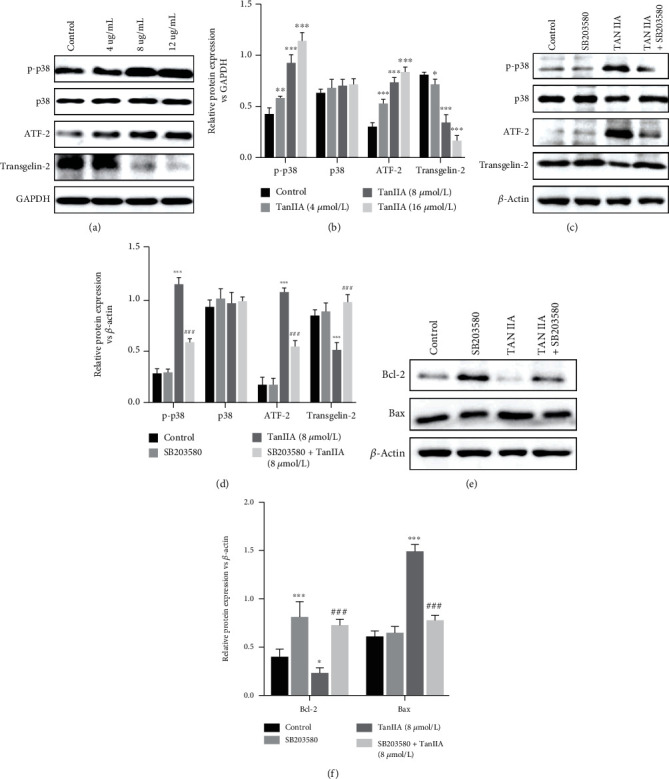
Effect of p38MAPK and ATF-2 expression in TanIIA induced- SW620 cells. Western blotting was used to ensure equal loading of proteins in each lane. (a–d) The ratio of p-p38MAPK, p38MAPK, ATF-2, and Transgelin-2 to GAPDH was calculated and expressed relative to that of control groups. (e, f) The expression of Bcl-2 and Bax was detected after different treatment (control, SB203580, TanIIA, and TanIIA+SB203580) of SW620 cells. ^∗^*P* < 0.05, ^∗∗^*P* < 0.01, ^∗∗∗^*P* < 0, 001compared with control group. ^###^*P* < 0.001, compared with TanIIA (8 *u*mol/L) group.

Studies have demonstrated that the expression of Transgelin-2 is positively associated with worse prognosis of different cancers [20]. Since the previous studies of us found that colorectal cancer cells SW620 apoptosis upon the activation of p38 MAPK, we made a point that whether Transgelin-2 expression relevant to the p38 MAPK activity. To investigate the mechanism of the apoptosis induction effect of TanIIA on SW620 cells, the expression of p-p38MAPK, p38MAPK, and ATF-2, Transgelin-2 were analyzed by Western blotting after treatment with TanIIA. As expected, the expression of p-p38MAPK and ATF-2 was upregulated, Transgelin-2 was downregulated by TanIIA in dose dependent pattern, whereas equal level of p38MAPK was revealed (Figure 5(a) and 5(b)). In order to verify the relationship between Transgelin-2 and p38MAPK, we then examined the expression level after treatment with p38MAPK specific inhibitor SB203580. Figures 5(c) and 5(d) showed that, p38MAPK inhibitor could reverse the downregulation of Transgelin-2, and same performance in p-p38MAPK and ATF-2. Additionally, the antiapoptotic protein Bcl-2 was significantly downregulated by TanIIA and also reversed with the treatment of SB203580, while the cellular induced apoptosis protein Bax was performed inversely (Figures 5(e) and 5(f)). All results supported that TanIIA could induce the apoptosis of SW620 cells via activating of p38MAPK signaling pathway which was involved with the downregulation of Transgelin-2.

## 4. Discussion

Apoptosis, a highly regulated form of cell death, defined by a series of morphological and biochemical changes involving the activation of cellular events, plays a significant role in preventing the development of cancer, and the damage of apoptosis is now considered to be a milestone in tumorigenesis [21, 22]. The activation of apoptosis pathway is a vital mechanism of cytotoxic drugs killing tumor cells [23]. Therefore, the induction of apoptosis has now been considered as a significant assessment method to evaluate the clinical efficacy of antineoplastic drugs and an important index for the selection of new antineoplastic drugs [24, 25].

Our study provides evidence for the potential anticancer effect of TanIIA on human CRC cell line SW620, which is achieved by inhibiting cell proliferation and inducing apoptosis. The results proved that TanIIA could inhibit tumor growth and extend the survival time on nude mice bearing transplanted colorectal tumor. TanIIA also reduced the growth of cell in a concentration- and time-dependent manner which was confirmed by CCK-8 assay. To prove whether cell death was caused by induced apoptosis, the Hoechst 33258 staining, FACS analysis, and propidium iodide staining were performed. The number of apoptotic nuclei with concentrated chromatin in the cells increased significantly after TanIIA treatment. The induction of apoptosis and/or the inhibition of cell proliferation is highly related to the activation of a variety of intracellular signal pathways to block the cell cycle in G0, G1, S, or G2-M phase. According to our results, it is speculated that TanIIA may induce obvious apoptosis of colorectal cancer cells and block the cells in G0/G1 phase in a concentration-dependent manner. In addition, TanIIA upregulated the expression of p-p38MAPK and ATF-2 by Western blot analysis.

p38 MAPK is a proline-directed serine/threonine kinase, which activated by multiple cellular stresses, including growth factors, lipopolysaccharide (LPS), inflammatory cytokines, ultraviolet, and osmotic shock [9, 26, 27]. Activating transcription factor 2 (ATF2) is the downstream signal of P38MAPK. When p38 is activated, p38-ATF2 binding increases, activating p38MAPK signal pathway [28]. Activated p38MAPK already proved that it could phosphorylate and activate MAPKAPK-2 and to phosphorylate the transcription factors Max, ATF-2, and MEF2 [6, 29]. p38 MAPK plays an important role in inflammation healing, tissue repair, embryonic development [30], immunoregulation [31], and the accommodation of cell proliferation and apoptosis [32]. Some studies reported that p38MAPK signal pathway proteins may be related to the regulation of apoptosis genes [33], or play a role in antitumor drugs activity [34]. Blockage of the p38MAPK pathway inhibits tumor cells apoptosis [35]. However, no study has examined the role p38MAPK plays in TanIIA–induced apoptosis in human colorectal tumor cells.

In order to investigate whether TanIIA induce apoptosis via p38MAPK signaling pathway, we assessed the apoptosis rate of cell and cell cycles after treatment with SB203580 and TSIIA by FACS analysis. The specific inhibitor of p38 MAPK–SB203580. This chemical inhibits MAPKAPK-2 activated by p38 MAPK and the followed HSP27's phosphorylation [36]. In our study, we found that after the block of p38MAPK signal pathway, the apoptosis rate of cells and the cell ratio of G0/G1 phase were decreased notably compared to with TanIIA. While they were no changed compared to control only treated with SB203580.

What is more, we observed that the cell-cell junction gradually disappeared, volume reduction and karyopyknosis with concentration gradient of TanIIA, which cause consideration of whether it is related to the alteration of cytoskeleton. Cytoskeleton not only plays a vital role in maintaining the shape of the cell, withstanding external forces, and maintaining the order of the internal structure of the cell but also participating in many significant life activities, including proliferation and apoptosis. Transgelin-2 is actin-binding protein, which involved in the constitution of cytoskeleton. In this study, we found Transgelin-2 regulated via p38 MAPK signaling in the colorectal cancer cells apoptosis by TanIIA.

In conclusion, our study confirmed that TanIIA has the anticancer effect and induce the apoptosis of CRC cells and block cells in G0/G1 phase, and we further demonstrated that this effect maybe mediated by p38MAPK signaling pathway and regulated the expression of Transgelin-2. These results demonstrated the potential of TanIIA as an antitumor drug is necessary to research and development of this new prospect.

## Data Availability

The data that support the findings of this study are available from the corresponding author upon reasonable request.

## Abbreviations

TanIIA: Tanshinone IIA
MAPK: Mitogen-activated protein kinase
CRC: Colorectal cancer
EMT: Epithelial-to-mesenchymal transition
PI: Propidium iodide
LPS: Lipopolysaccharide.
